# Reduced Muscle Force in Dystrophic *DMD*Δ52 Pigs Is Incompletely Restored by Systemic Transcript Reframing (*DMD*Δ51–52)

**DOI:** 10.1002/jcsm.70084

**Published:** 2025-10-01

**Authors:** Michaela Blasi, Hristiyan Hristov, Jan B. Stöckl, Martin Kraetzl, Sonja Fiedler, Elisabeth Kemter, Mayuko Kurome, Barbara Kessler, Josep M. Cambra, Valeri Zakhartchenko, Maggie C. Walter, Christian Kupatt, Nikolai Klymiuk, Thomas Fröhlich, Andreas Blutke, Michael Stirm, Florian Jaudas, Eckhard Wolf

**Affiliations:** ^1^ Chair of Molecular Animal Breeding and Biotechnology, Gene Center and Department of Veterinary Sciences LMU Munich Munich Germany; ^2^ Center for Innovative Medical Models (CiMM) LMU Munich Munich Germany; ^3^ Laboratory for Functional Genome Analysis (LAFUGA), Gene Center LMU Munich Munich Germany; ^4^ Institute of Veterinary Pathology, Center for Clinical Veterinary Medicine LMU Munich Munich Germany; ^5^ German Center for Child and Adolescent Diseases (DZKJ) Munich Germany; ^6^ Klinik und Poliklinik für Innere Medizin I TUM Klinikum Rechts der Isar Munich Germany; ^7^ Friedrich Baur Institute at the Department of Neurology LMU University Hospital, LMU Munich Munich Germany; ^8^ German Center for Cardiovascular Research (DZHK) Munich Heart Alliance Munich Germany; ^9^ Interfaculty Center for Endocrine and Cardiovascular Disease Network Modelling and Clinical Transfer (ICONLMU) LMU Munich Munich Germany

**Keywords:** Becker muscular dystrophy, Duchenne muscular dystrophy, dystrophin, muscle force, pig model

## Abstract

**Background:**

Duchenne muscular dystrophy (DMD) is a fatal X‐linked disease caused by mutations in the *DMD* gene, leading to dystrophin deficiency and progressive degeneration of skeletal and cardiac muscles. Pigs lacking *DMD* exon 52 (*DMD*Δ52) are a clinically severe model for DMD, mimicking molecular, functional and pathological hallmarks of the human disease. Dystrophin expression can be restored by additionally deleting exon 51 (*DMD*Δ51–52), which reframes *DMD* transcripts and alleviates pathological alterations. *DMD*Δ51–52 pigs model Becker muscular dystrophy (BMD), a milder and slower‐progressing form of muscle degeneration.

**Methods:**

The Aurora Swine Isometric Footplate Test Apparatus was used to quantify functional parameters of the hind limb dorsiflexor muscle group of 3.5‐month‐old DMD pigs (*n* = 5), BMD pigs (*n* = 3) and wild‐type (WT) littermate controls (*n* = 3). In addition, histopathological and proteomic studies of the functionally tested muscles were performed.

**Results:**

After twitch stimulation, DMD muscle achieved 62.4% (*p* < 0.05) and BMD muscle 67.1% (*p* < 0.05) of the absolute peak force of WT muscle, indicating partial but not complete restoration of muscle force by *DMD*Δ51–52 transcript reframing. After normalization to the cube root of body mass, the values were 70.9% for DMD muscle (*p* = 0.05) and 65.8% for BMD muscle (*p* < 0.05). DMD muscle showed a reduced rate of contraction (*p* < 0.01); the rate of relaxation was decreased in both DMD (*p* < 0.01) and BMD (*p* < 0.05) compared with WT muscle. After tetanic stimulation, DMD muscle reached 54.7% (*p* < 0.001) and BMD muscle 80.4% (*p* = 0.08) of WT muscle force. Normalized values were 62.7% (DMD; *p* < 0.01) and 79.3% (BMD; *p* = 0.08). The rate of contraction was reduced in both DMD (*p* < 0.001) and BMD muscle (*p* < 0.01), whereas the return to the resting state was prolonged (*p* < 0.001) only in DMD vs. WT muscle. Histopathology and proteomics revealed no significant differences between BMD and WT muscles, whereas severe alterations were observed in DMD pigs.

**Conclusions:**

Our study pioneers the quantitative assessment of skeletal muscle function in dystrophic pigs. It demonstrates that systemic exon 51 skipping in DMD caused by loss of *DMD* exon 52 partially restores muscle function but does not achieve WT levels. These findings highlight the value of dynamic muscle force measurements as a sensitive tool to evaluate the efficacy of therapeutic interventions in the porcine DMD model.

## 1 Introduction

Duchenne muscular dystrophy (DMD) is an X‐linked neuromuscular disorder affecting approximately one in every 5000–6000 male births [1]. The disease is caused by disruption of the open reading frame (ORF) of the dystrophin (*DMD*) gene, one of the largest protein‐coding genes in the mammalian genome, leading to the absence of functional dystrophin, a protein essential for stabilizing muscle fibres [2]. The lack of dystrophin results in muscle fibre damage, pathological calcium influx, muscle weakness, loss of ambulation, the need for respiratory support and ultimately failure of respiratory and cardiac function leading to premature death [3, 4].

Animal models have played key roles in dissecting disease mechanisms of DMD and in developing and testing new diagnostic procedures and therapeutic concepts [5]. The *mdx* mouse model, as well as genetically engineered mouse strains resembling human *DMD* mutations [6], is most widely used but has limitations in the resemblance of the human DMD phenotype. Moreover, the small size of rodent models limits the translatability of positively tested therapeutic approaches to human patients. Therefore, large animal models are essential for bridging the gap to clinical studies [7]. Dog models with spontaneous *DMD* mutations, most prominently the Golden Retriever muscular dystrophy (GRMD) [8], but also other canine models (reviewed in [9]), have been successfully used for this purpose (e.g., [10, 11, 12]). In the last decade, several pig models for DMD have been developed to complement the existing canine models and have several advantages: (i) Human *DMD* mutations can be reproduced by genetic engineering/gene editing; (ii) the rapid muscle growth of pigs leads to a highly progressive muscular dystrophy; (iii) cardiac involvement is visible from an early age; (iv) because of the favourable reproductive performance of pigs, the establishment of test cohorts for nonclinical studies is straightforward (reviewed in [13]).

We have developed a porcine DMD model lacking *DMD* exon 52 (*DMD*Δ52), which resembles a frequent human *DMD* mutation and reveals molecular, clinical and pathological hallmarks of the human disease [14, 15]. Compared with DMD patients, the disease progression in *DMD*Δ52 pigs is markedly accelerated, which allows safety and efficacy readouts for new treatment strategies in a reasonable period of time. *DMD*Δ52 pigs have been used for studying disease mechanisms by omics‐profiling of skeletal muscle [14, 15, 16] and heart samples [15, 17] and for validating multispectral optoacoustic tomography (MSOT) as a tool for non‐invasive monitoring of muscle fibrosis [18]. In addition, the model has been used for testing a somatic gene editing approach to restore the disrupted *DMD* reading frame in *DMD*Δ52 pigs, which results in the complete absence of dystrophin. Adeno‐associated viral vectors encoding the two parts of a split‐Cas9 system and 2 guide RNAs binding upstream or downstream of *DMD* exon 51 were applied intramuscularly or intravenously, resulting in *DMD*Δ51–52 genetic events and the production of an internally shortened but apparently functional dystrophin [19]. Consequently, the locomotor activity as well as biochemical and pathological parameters of the systemically treated *DMD*Δ52 pigs were improved. In order to predict the best possible therapeutic outcome of the exon 51 deletion approach in DMD caused by the loss of exon 52, we engineered pigs lacking *DMD* exons 51 and 52 (*DMD*Δ51–52) in every cell [20]. In these pigs, dystrophin was restored, clinical‐chemical parameters and cardiac function were normalized, and the skeletal muscle pathology was ameliorated. Therefore, *DMD*Δ51–52 pigs resemble Becker muscular dystrophy (BMD), which is caused by *DMD* mutations with an intact reading frame and results in a later onset and a milder disease phenotype compared with DMD, as internally truncated but partially functional dystrophin is still produced [2].

In the present study, we expand the characterization of our *DMD*Δ52 and *DMD*Δ51–52 pig models by a quantitative in vivo assessment of skeletal muscle function using a system that has been previously employed for the characterization of GRMD dogs [21] and WT pigs [22]. The Aurora A892 Swine Isometric Footplate test device allows precise and reproducible evaluation of intrinsic muscle functions by stimulating the dorsal flexors of the hind limbs using electric impulses. The resulting contractions and consequent movements of the hind limb are measured by a sensor pedal attached to the hind limb [22, 23]. Through this, we performed a comprehensive characterization of muscle force and endurance, revealing a massive impairment of muscle functions in DMD pigs (*DMD*Δ52). Although muscle strength was markedly improved in BMD (*DMD*Δ51–52) compared with DMD pigs, it was not fully restored to the level of WT pigs. Further characterization of the functionally tested muscles was performed by histological and proteomic investigations.

## 2 Materials and Methods

### 2.1 Generation of the Study Cohort

**FIGURE 1:**
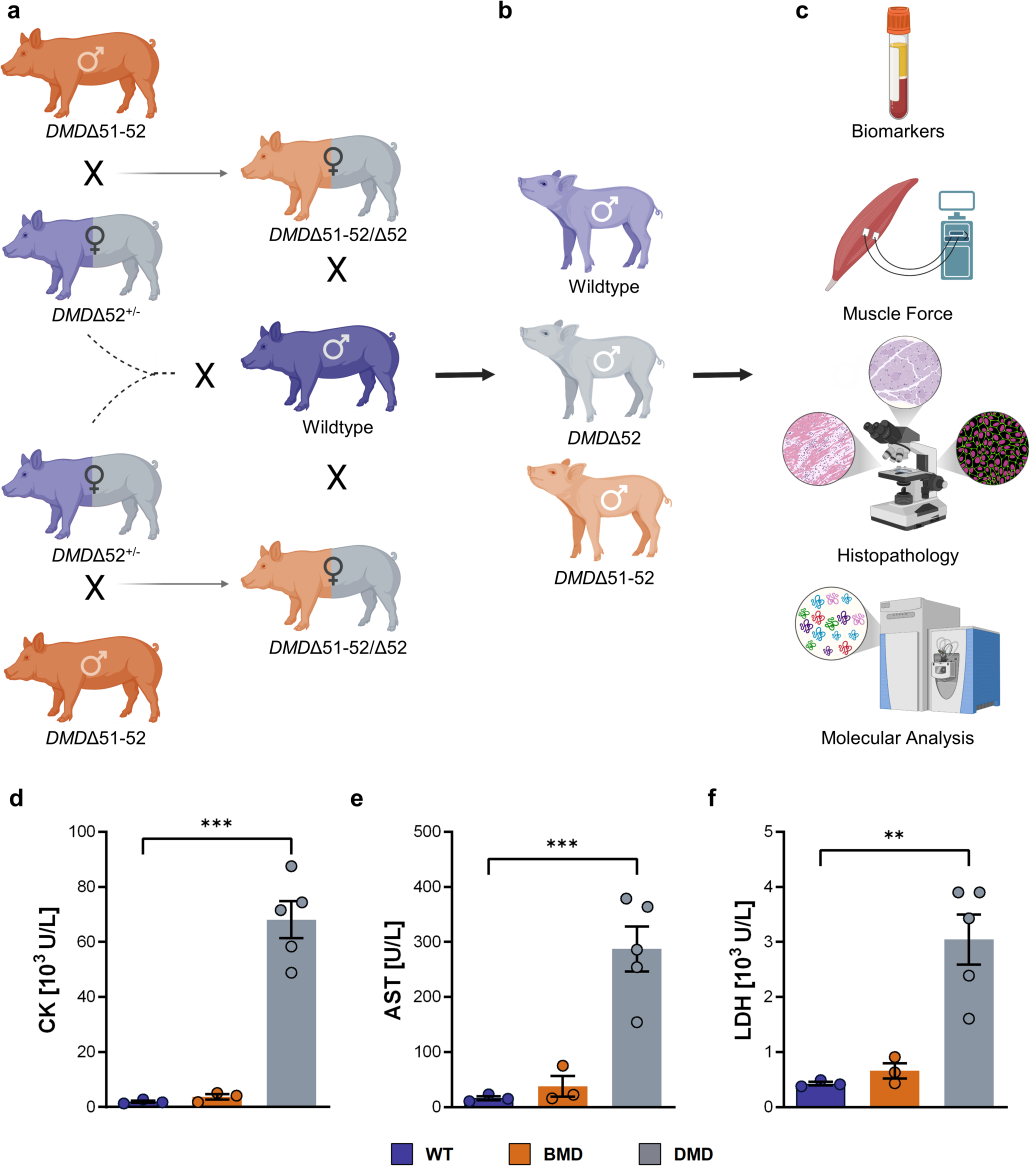
Study cohort and design (a‐c) Schematic representation of animal production and experimental analysis. Two cloned BMD (*DMD*Δ51–52) boars were bred with two *DMD*Δ52 carrier sows. The two carrier sows, as well as two female offspring carrying one *DMD*Δ51–52 and one *DMD*Δ52 allele, were mated with the same wild‐type (WT) boar to produce the experimental animals for the present study (a). The resulting offspring included male WT (*n* = 3), DMD (*DMD*Δ52; *n* = 5), and BMD (*DMD*Δ51–52; *n* = 3) pigs (b). In 3.5‐month‐old pigs, analyses of biochemical blood parameters, muscle function measurements and histopathological investigations and molecular analysis of skeletal muscle were performed (c). (d‐f) Serum activities of enzymes indicative of muscle damage: creatine kinase (CK) (d), aspartate aminotransferase (AST) (e), and lactate dehydrogenase (LDH) (f). Means ± SEM and values of individual animals are shown. Statistical analysis was performed using one‐way ANOVA with Dunnett's post hoc test, comparing the BMD and DMD groups with the WT control group. Significant differences are indicated by asterisks (**p < 0.01; ***p < 0.001).

Two BMD boars (*DMD*Δ51–52) were bred to heterozygous *DMD*Δ52 carrier sows. These two carrier sows, as well as two female offspring carrying one *DMD*Δ51–52 and one *DMD*Δ52 allele, were mated with a single WT boar to produce the male DMD, BMD and WT pigs investigated in this study (Figure 1a,b). During the first 2 weeks after birth, extensive care for the DMD piglets was performed to ensure minimal mortality as described previously [20]. The genotyping of the piglets was performed by PCR detection of the loss of *DMD* exon 51 (BMD) or 52 (DMD). A cohort of 3.5‐month‐old DMD (*n* = 5), BMD (*n* = 3) and WT (*n* = 3) pigs (full‐ or half‐siblings) was investigated in this study.

### 2.2 Muscle Force Measurements

**FIGURE 2:**
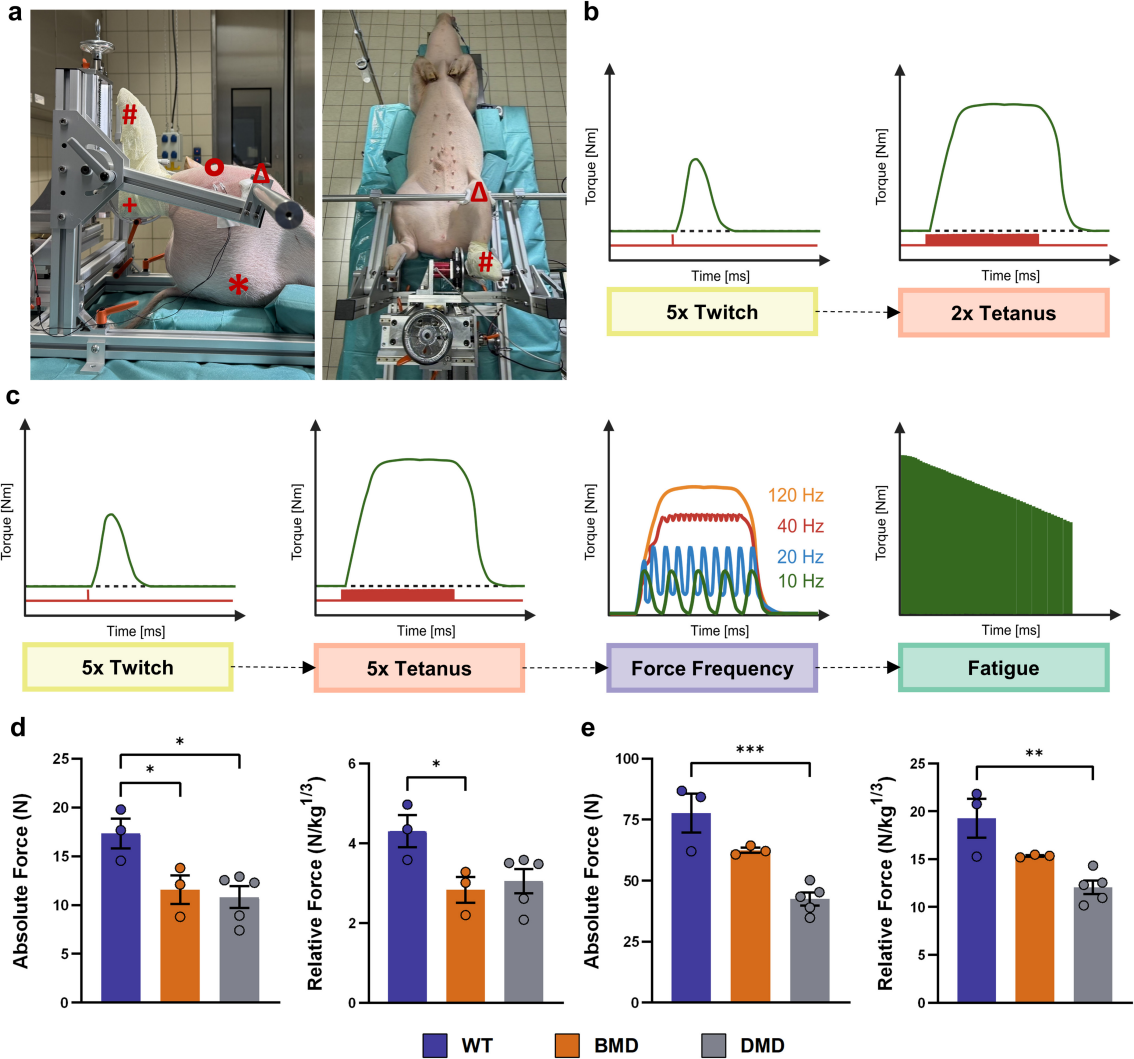
Experimental protocol and peak force measurements (a) Muscle function assessments were conducted in anaesthetized pigs on the dorsiflexor muscle group of the hindlimb, including the *Mm. tibialis cranialis*, *fibularis tertius*, *fibularis longus* and *fibularis brevis* for flexion of the hock joint and *Mm. extensor digitorum longus*, *extensor digitorum lateralis* and *extensor hallucis longus* for the extension of the tip of the claw. These assessments were performed according established procedures [22, 24, 25] using a commercially available integrated Swine Isometric Footplate Test Apparatus (892A, Aurora Scientific). To ensure proper biomechanical alignment, the hip (*), knee (Δ) and hock joints (+) were fixed at a 90° angle, while the sole was fixed on a footplate connected to a sensitive torque transducer (#), providing an optimal physiological configuration for maximum peak force generation. Electrodes (Ο) were positioned to electrically stimulate the *N. fibularis*, with stimulation current individually optimized to achieve maximum torque amplitude in response to a single electronic nerve stimulus, ensuring selective stimulation of the dorsiflexors while avoiding activation of the plantarflexor muscles. The applied stimulation current ranged from 65 to 400 mA, depending on the individual animal's response. After ensuring correct electrode positioning, knee fixation and selecting the appropriate stimulation amplitude, (b) 5 twitch stimulations (0.2 ms pulse width, 15 s resting in between) and 2 tetanic stimulations (100 Hz, 500 ms stimulation period, 15 s resting in between) were carried out to check the correct fit of the electrode. (c) After a resting period of 3 min, the measurements for data collection were started: 5 twitch stimulations, 5 tetanic stimulations and force frequency measurements (8 stimulations between 10–120 Hz, each 500 ms stimulation period with 60 s resting in between) assessed the relationship between stimulation frequency and torque. Subsequent fatigue testing involved repeated 100 Hz tetanic contractions over 10 min, demonstrating torque decline with muscle fatigue. Further details on stimulation durations, frequencies, intervals and resting periods are provided in Table S1. (d) Peak force following a single stimulus (twitch) displayed as absolute force (left panel) and relative force (normalized to cube root of body mass; right panel). (e) Peak force following tetanic stimulation. The arrangement of the graphs follows the layout presented in Panel d. Means ± SEM and values of individual animals are shown. Statistical analysis was performed using one‐way ANOVA with Dunnett's post hoc test, comparing the BMD and DMD groups with the WT control group. Significant differences are indicated by asterisks (*p < 0.05; **p < 0.01; ***p < 0.001).

For the examination of muscle physiology, the 892A Swine Footplate Test Apparatus by Aurora Scientific combined with a Monitor (22″ Widescreen LCD [608C]) and High‐Power Follow Stimulator (701C) was used. All animals underwent general anaesthesia using ketamine (Ursotamin, Serumwerk Bernburg), azaperone (Azaporc, Serumwerk Bernburg) and xylazine (Xylazin, Serumwerk Bernburg) and were positioned supine on a table to which the Aurora system was attached. Adequate anaesthesia maintenance was performed throughout the whole procedure, including pulse oximetry for oxygen saturation and heart rate (LifeVet PT Pulsoximeter, Eickenmeyer), monitoring of respiratory rate and body temperature (Microlife) as well as reflex control at appropriate intervals. The measurement protocol is visualized in Figure 2a–c and Table S1. Data analysis was performed using the 611A Dynamic Muscle Analysis (DMAv5.501) software (Aurora Scientific). The relative force was obtained by dividing the measured absolute force by the cube root (to keep the same dimension) of body mass. This ensures that observed differences reflect true physiological effects rather than just size differences.

### 2.3 Clinical Chemistry

Blood samples were collected under general anaesthesia from the right jugular vein using Serum Monovettes (Sarstedt). After allowing the blood to clot at room temperature for 30 min, serum was separated by centrifugation at 1800 *g* for 20 min at 4°C. The separated serum was then stored at −80°C until further processing. The serum activities of creatine kinase (CK), lactate dehydrogenase (LDH) and aspartate aminotransferase (AST) were determined by an external large animal veterinary laboratory as described previously [15].

### 2.4 Histopathology

Fully anaesthetized animals (see above) were euthanized by intravenous injection of T 61 (Intervet). Tissue specimens from the skeletal muscle group of interest (including the *Musculus tibialis cranialis*, *Musculus fibularis tertius* and *Musculus fibularis longus*) were systematically sampled as described previously [26]. These tissue samples were formalin‐fixed and routinely embedded in paraffin. Sections of 3‐μm thickness were stained with haematoxylin–eosin and Sirius red. The stained sections were examined using an Olympus BX 43 light microscope, and digital images were acquired at 20× objective magnification with a digital camera (DP 72, Olympus) and adapted software (VISVisiopharm Integrator System Version 3.4.1.0, Visiopharm A/S). The samples for histopathological investigation were not blinded to the investigator. For each animal, 10 randomized fields of view at 40× objective magnification were recorded to examine the position of the cell nuclei and Feret diameter of muscle fibres. The proportion of collagen was analysed using the ImageJ software (version 1.53 k).

### 2.5 Immunohistochemistry

Immunohistochemical (IHC) detection of cellular dystrophin abundance patterns was carried out as previously described [20]. Briefly, 3‐μm‐thick sections of distal hindlimb muscle were incubated with monoclonal mouse anti‐DYS1 (rod domain; NCL‐DYS1, clone Dy4/6D3, Leica Biosystems; dilution 1:20, overnight at 4°C) and after thorough washing with biotinylated goat antimouse IgG (no. 115‐065‐146, lot 118 375, Jackson ImmunoResearch; dilution 1:500, 1 h at room temperature). Appropriate negative control sections (omission of primary antibody) were used. Bound antibodies were detected using the Vectastain Elite ABC HRP kit with 3.3′‐diaminobenzidine tetrahydrochloride dihydrate as chromogen (brown colour). Meyer's hemalum was applied as a counterstain. Stained sections were photographed at 20× objective magnification using an Olympus BX 43 light microscope and digital camera (DP 72, Olympus) and adapted software (VISVisiopharm Integrator System Version 3.4.1.0, Visiopharm A/S).

### 2.6 Immunofluorescence

Immunofluorescence staining was performed using paraffin‐embedded samples of the fibularis tertius muscle, sectioned at 3‐μm thickness. Tissue sections were blocked for 1 h at room temperature in 5% normal donkey serum (NDS) diluted in PBS to prevent nonspecific binding. Primary antibody incubation was carried out overnight at 4°C using the antislow myosin heavy chain (MYH7, 22 280‐1‐AP, Proteintech, dilution 1:400) and antifast myosin heavy chain (MHC fast, Leica NCL‐MHCf, dilution 1:100) antibodies. After washing with PBS, sections were incubated for 1 h at room temperature with the corresponding secondary antibodies: AlexaFluor Plus 555‐conjugated donkey antirabbit IgG (H + L) (A32794, Invitrogen, dilution 1:1000) and AlexaFluor Plus 488‐conjugated donkey antimouse IgG (H + L), highly cross‐adsorbed (A32766, Invitrogen, dilution 1:700).

Following additional PBS washes, nuclei were stained with DAPI (1:4000 in PBS). To reduce background fluorescence, the Vector TrueVIEW Autofluorescence Quenching Kit (SP‐8400, Vector Laboratories) was applied according to the manufacturer's instructions. After a final PBS wash, sections were mounted and images were acquired using a confocal fluorescence microscope (Leica SP8 Confocal).

### 2.7 Proteomics and Targeted Mass Spectrometry

Samples for proteomics were prepared as previously described [20]. In brief, skeletal muscle samples were cryo‐pulverized, lysed aided by sonication and digested with Lys‐C and trypsin. Aliquots of 800‐ng peptides were spiked with 80 fmol of synthetic heavy peptides (JPT). Peptides were trapped on a PepMap Neo trap column (C18, 5 mm × 300 μm, 5 μm; Thermo Fisher Scientific) and then separated on a PepSep Ultra analytical column (C18, 25 cm × 75 μm, 1.5 μm; Bruker) using a nanoElute 2 liquid chromatography system (Bruker). Mass spectrometry was performed with a timsTOF HT (Bruker) in data‐independent acquisition mode. Raw data were searched with DIA‐NN 2.1.0 [27] and the UniProt *Sus scrofa* reference proteome (UP000008227, retrieval date: 10/2024). For the direct quantification of dystrophin using the heavy labelled peptides, Skyline 24.1.0.199 was used [28]. Data analysis and visualization were done with Perseus 1.6.7.0 [29] and R 4.4.1. The mass spectrometry proteomics data have been deposited to the ProteomeXchange Consortium via the PRIDE [30] partner repository with the dataset identifier PXD064879.

### 2.8 Statistical Analysis

Statistical analyses were performed using Prism 10 software (GraphPad). Gaussian distribution was assessed using the Shapiro–Wilk test. As the data followed a normal distribution, statistical significance was determined using a one‐way analysis of variance (ANOVA) with Dunnett's post hoc test. A *p*‐value of < 0.05 was considered statistically significant. For the whole proteome analysis, the false discovery rate of identified proteins was controlled to be 1%. To test for differentially abundant proteins, a modified *t*‐test was employed, allowing the computation of permutation‐based cut‐off curves that control the false discovery rate to 5%.

### 2.9 Ethical Approval

All animal experiments were conducted according to the German Animal Welfare Act with the permission of the responsible regulatory authority (Government of Upper Bavaria; AZ 55.2‐2532.Vet_02‐19‐195 and AZ 55.2‐2532.Vet_02‐22‐92).

## 3 Results

### 3.1 Establishment of a Standardized Cohort to Evaluate the Effects of *DMD* Mutations on Muscle Function

To standardize the genetic background of the experimental groups, two cloned BMD (*DMD*Δ51–52) boars [20] were mated with two DMD (*DMD*Δ52) carrier sows [15]. The two carrier sows, as well as two female offspring carrying one *DMD*Δ51–52 and one *DMD*Δ52 allele, were mated with a single WT boar (Figure 1a). The resulting male DMD, BMD and WT piglets used in the present study were thus full‐ or half‐siblings (Figure 1b). At the age of 3.5 months, serum biomarkers were analysed and in vivo measurements of muscle function were performed, followed by histopathological and molecular assessments of the stimulated muscle region after necropsy in order to further characterize the muscle phenotype (Figure 1c). Although muscle damage‐related serum markers, including creatine kinase (CK), aspartate aminotransferase (AST) and lactate dehydrogenase (LDH) activities, were significantly elevated in DMD pigs, they were similar to WT levels in BMD pigs (Figure 1d–f).

### 3.2 Reduced Muscle Strength in DMD and BMD Pigs

To further evaluate the consequences of absent or truncated dystrophin, we performed neural‐evoked functional assessment of skeletal musculature. Using the Swine Isometric Footplate Test Apparatus (Figure 2a), muscle strength was assessed following proper biomechanical alignment. Standardized measurement protocols were applied (Figure 2b–c and Table S1). To account for growth retardation observed in DMD pigs (Figure S1), the measured force was normalized to the cube root of body mass [22, 24, 25]. Furthermore, muscle strength was evaluated using two electro‐neural stimulation modes: a single stimulus (twitch) and a tetanic stimulus, with the latter inducing a stronger muscle response. After twitch stimulation, BMD pigs achieved 67.1% (mean ± SEM: 11.6 ± 1.5 N; *p* < 0.05) and DMD pigs 62.4% (10.8 ± 1.1 N; *p* < 0.05) of the absolute peak force observed in WT littermates (17.3 ± 1.5 N; Figure 2d, left). After normalization, the relative force measured in the BMD group reached 65.8% (2.8 ± 0.3 N/kg^1/3^; *p* < 0.05), whereas DMD pigs achieved 70.9% (3.1 ± 0.3 N/kg^1/3^; *p* = 0.05) of their WT littermates' force (4.3 ± 0.4 N/kg^1/3^; Figure 2d, right).

To determine the stimulation frequency at which individual twitch contractions summate into a fully fused tetanic contraction, a force‐frequency protocol was conducted across all three genotypes. The analysis revealed that stimulation at 100 Hz resulted in complete summation of single twitch peaks into a sustained tetanic plateau in all genotypes (Figures S2 and S3). Notably, after tetanic stimulation, the DMD muscle displayed a marked decrease in force, corresponding to 54.7% (42.5 ± 2.7 N; *p* < 0.001) of the absolute force observed in WT (77.7 ± 7.9 N). BMD muscle strength was less decreased, reaching 80.4% (62.5 ± 1.1 N; *p* = 0.08) of the absolute force of WT muscle (Figure 2e, left). After normalization for the cube root of body mass, the deficit in muscle strength remained, with DMD pigs reaching 62.7% (12.1 ± 0.7 N/kg^1/3^; *p* < 0.01) and BMD pigs 79.3% (15.3 ± 0.1 N/kg^1/3^; *p* = 0.08) of the force detected in WT pigs (19.3 ± 2.0 N/kg^1/3^; Figure 2e, right).

### 3.3 Decline in Muscle Dynamics in DMD Pigs and Recovery in BMD Pigs

**FIGURE 3:**
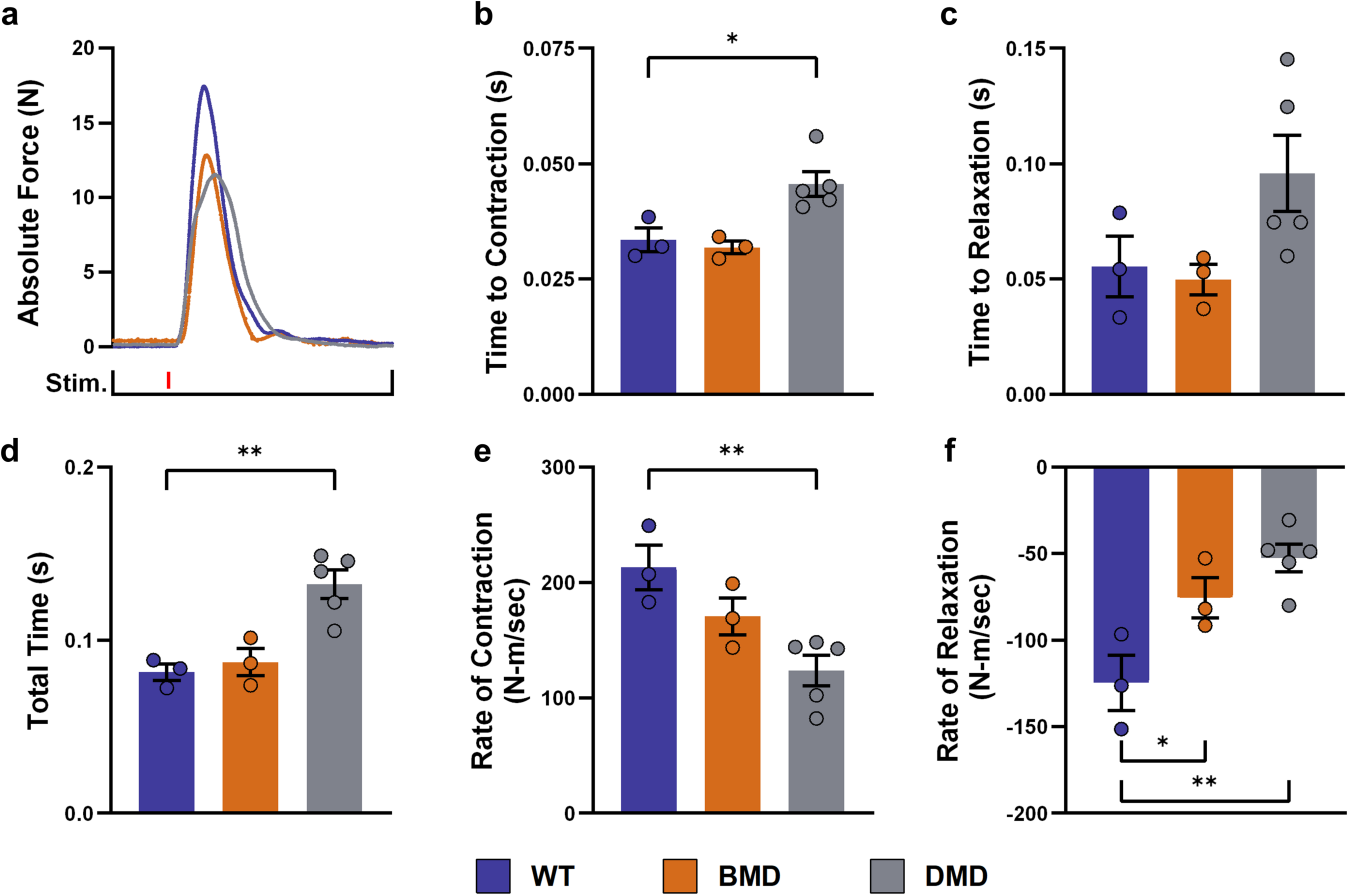
Muscle dynamics in DMD, BMD, and WT pigs during twitch contractions (a) Representative illustration of original trajectories. Time to reach peak force from baseline (b), time to 95% relaxation (c) and total duration (d) of a twitch contraction. Maximum contraction (e) and relaxation (f) rates during the twitch protocol. Means ± SEM and values of individual animals are shown. Statistical analysis was performed using one‐way ANOVA with Dunnett's post hoc test, comparing the BMD and DMD groups with the WT control group. Significant differences are indicated by asterisks (*p < 0.05; **p < 0.01).

Evaluation of skeletal muscle function not only revealed reduced muscle strength but also profound abnormalities in the dynamics of DMD muscle. Following twitch excitation (Figure 3a), the contraction phase was significantly prolonged in DMD vs. WT muscle (0.045 ± 0.0027 s vs. 0.030 ± 0.0025 s; *p* < 0.05) (Figure 3b), and also, the relaxation phase was, as a trend, prolonged (Figure 3c), resulting in a notably prolonged total duration of the contraction in DMD (0.13 ± 0.008 s; *p* < 0.01) vs. WT muscle (0.080 ± 0.004 s) (Figure 3d). Consequently, DMD muscle revealed significantly decreased contraction (124 ± 13.3 N‐m/s vs. 213 ± 19.3 N‐m/s in WT; *p* < 0.01) and relaxation slopes (−52.5 ± 7.99 N‐m/s vs. −125 ± 15.8 N‐m/s in WT; *p* < 0.01) (Figure 3e,f). Although contraction and relaxation phases, as well as the total duration of the contraction, were not different between BMD and WT muscle, the relaxation slope of BMD muscle (−75.4 ± 11.7 N‐m/s) was—due to the lower peak force—significantly (*p* < 0.05) reduced compared with WT muscle (Figure 3f).

**FIGURE 4:**
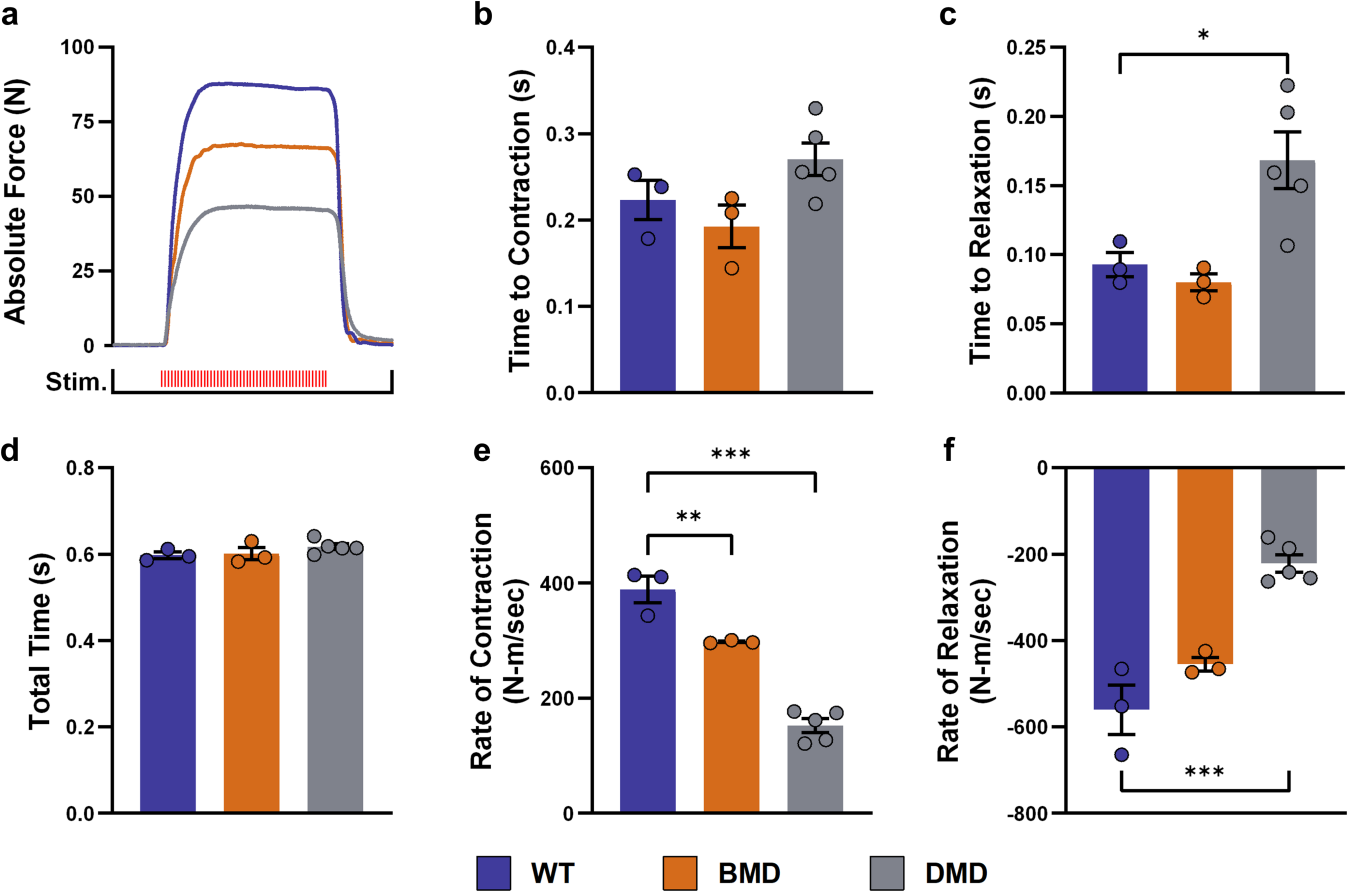
Muscle dynamics in DMD, BMD, and WT pigs during tetanic contractions (a) Representative illustration of original trajectories. Time to reach peak force from baseline (b), time to 95% relaxation (c) and total duration (d) of a tetanic contraction. Maximum contraction (e) and relaxation (f) rates during the tetanic protocol. Means ± SEM and values of individual animals are shown. Statistical analysis was performed using one‐way ANOVA with Dunnett's post hoc test, comparing the BMD and DMD groups with the WT control group. Significant differences are indicated by asterisks (*p < 0.05; **p < 0.01; ***p < 0.001).

During tetanic stimulations (Figure 4a), alterations in muscle dynamics were less pronounced. Nevertheless, the return to the resting state remained significantly prolonged in DMD vs. WT muscle (0.17 ± 0.02 s vs. 0.093 ± 0.0089 s; *p* < 0.05) (Figure 4c). Because of a smaller peak force, the slope of contraction was significantly decreased in both DMD (152 ± 11.8 N‐m/s; *p* < 0.001) and BMD muscle (298 ± 1.44 N‐m/s; *p* < 0.01) compared with WT (389 ± 23 N‐m/s) muscle (Figure 4e). In addition, the relaxation slope was significantly (−221 ± 20.2 N‐m/s; *p* < 0.001) decreased in DMD vs. WT muscle (−560 ± 57.6 N‐m/s) (Figure 4f).

Next, we aimed to evaluate muscle endurance through a series of 100 consecutive tetanic stimulations, assessing the decline in peak force over time. Although technical difficulties during initial measurements limited data availability and statistical power, a consistent trend was observed. Despite starting from different initial force levels, DMD pigs (*n* = 3) exhibited a more rapid decline (Figure S4a), reaching 75% of the maximum force already after 29 stimulations, whereas BMD (*n* = 2) and WT pigs (*n* = 2) required nearly twice as many stimulations to reach the same 75% force threshold (Figure S4b), indicating a faster onset of fatigue in DMD muscle.

### 3.4 Dystrophy of Distal Hind Limb Dorsiflexor Muscles in DMD but Not BMD Pigs

**FIGURE 5:**
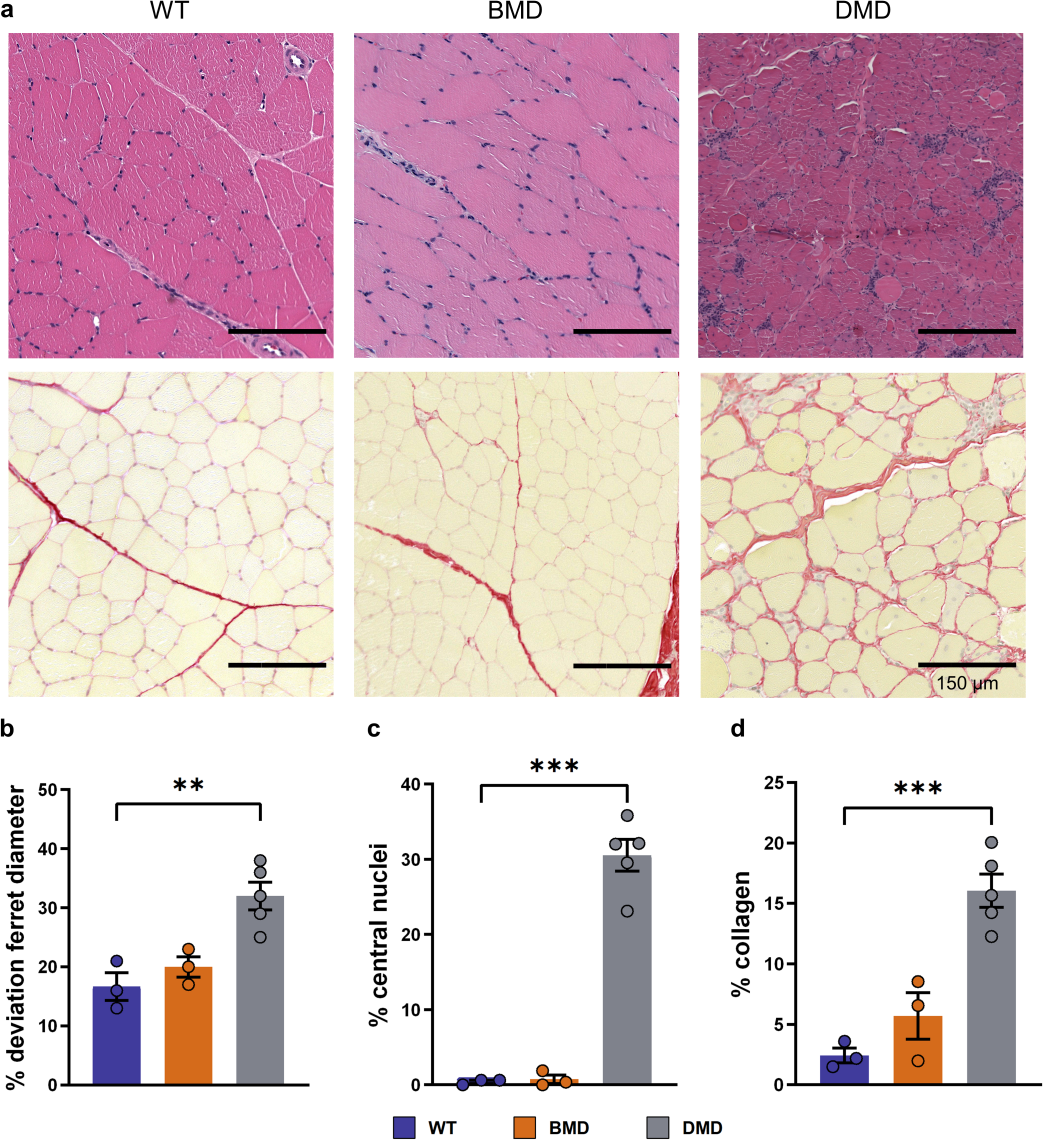
Histopathological examination of stimulated muscles. (a) Representative images of histologic sections of the *M. fibularis tertius* from WT, BMD and DMD pigs after staining with haematoxylin–eosin (upper panel) and Sirius red (lower panel). (b‐d) Quantification of histological alterations. Graphical representation of the percentage deviation of the Ferret diameter (b), the percentage of the central nuclei (c) and the percentage of collagen (d) of the stimulated muscle. Mean ± SEM and values of individual animals are shown. Statistical analysis was performed using one‐way ANOVA with Dunnett's post hoc test, comparing the BMD and DMD groups with the WT control group. Significant differences are indicated by asterisks (**p < 0.01; ***p < 0.001).

Similar to other muscle groups [20], the distal hind limb muscles of DMD pigs exhibited prominent histopathological abnormalities. Haematoxylin–eosin and Sirius red staining revealed inhomogeneous muscle tissue and an increased presence of immune cell infiltrates, indicating inflammation (Figure 5a). Muscle fibre diameters in DMD pigs displayed a large variation. Analysis of the Feret's diameter revealed a significantly higher variance in DMD pigs (32.0% ± 2.4%; *p* < 0.01) compared with BMD (20.0% ± 1.7%) and WT pigs (16.7% ± 2.3%) (Figure 5b), consistent with the disrupted muscle fibre integrity characteristic of dystrophic muscle. A high frequency of muscle fibres with centrally located nuclei, a hallmark of dystrophic pathology, was evident in DMD pigs. In contrast, WT and BMD pigs predominantly displayed peripherally located nuclei, consistent with healthy muscle morphology. Quantification showed that 30.5% ± 2.1% (*p* < 0.001) of muscle fibres in DMD pigs had centrally located nuclei, compared with only 0.75% ± 0.57% in BMD pigs and 0.42% ± 0.20% in WT pigs, with no significant differences between the BMD and WT groups (Figure 5c). Additionally, collagen content, a marker of fibrosis, measured by digital image analysis, was significantly elevated in DMD muscle, reaching 16.1% ± 1.4% (*p* < 0.001), compared with 5.7% ± 1.9% in BMD and 2.4% ± 0.6% in WT muscle (Figure 5d). These histological findings underscore that severe structural abnormalities are present in distal hind limb muscles of DMD pigs, whereas the muscle phenotype of BMD pigs closely resembled that of WT pigs.

### 3.5 Altered Muscle Fibre Type Composition in DMD, but Not BMD Muscle

**FIGURE 6:**
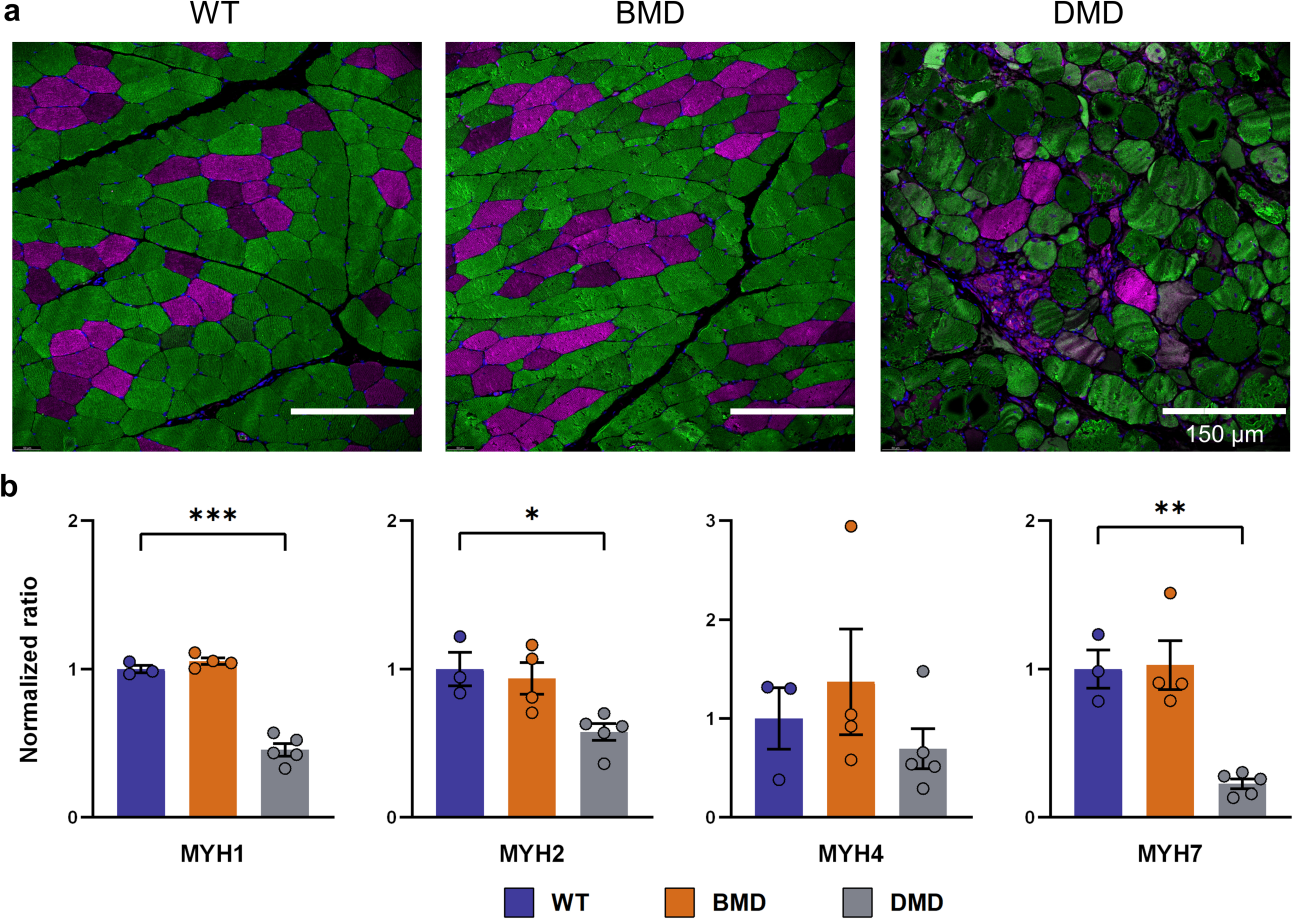
Muscle fibre typing based on myosin heavy chain isoforms (a) Representative immunofluorescence images of cross‐sections of the *M. fibularis tertius* in WT, BMD, and DMD samples, stained with antibodies against slow myosin heavy chain (magenta) and fast myosin heavy chain (green). (b) Quantification of selected myosin heavy chain isoforms using mass spectrometry. The ratios were normalized to the mean of WT levels. Mean ± SEM and values of individual animals are shown. Statistical analysis was performed using one‐way ANOVA with Dunnett's post hoc test, comparing the BMD and DMD groups with the WT control group. Significant differences are indicated by asterisks (*p < 0.05; **p < 0.01; ***p < 0.001).

Immunofluorescence‐based fibre typing further revealed abnormalities in the muscle fibre composition of DMD samples. Analysis of the *M. fibularis tertius* demonstrated a substantial reduction in slow‐twitch (Type I) fibres in DMD compared with BMD and WT samples. BMD and WT samples revealed a similar fibre distribution (Figure 6a). This observation was supported by the results of a holistic proteome analysis of the muscle samples. An overview is presented in Figure S5. Figure 6b shows the quantification of relevant myosin heavy chain (MYH) isoforms. MYH7, a slow‐twitch isoform, was markedly downregulated in DMD muscle. Additionally, MYH1 and MYH2, associated with fast‐twitch Type IIx and Type IIa fibres, respectively, were significantly reduced in DMD compared with WT muscle samples. In contrast, the levels of MYH4, characteristic of the Type IIb fibres, did not differ significantly between the DMD and WT groups. No significant differences in the abundances of MYH isoforms were noted between BMD and WT samples.

### 3.6 Near Normal Dystrophin Complex Protein Profiles in BMD Dorsiflexor Muscles

**FIGURE 7:**
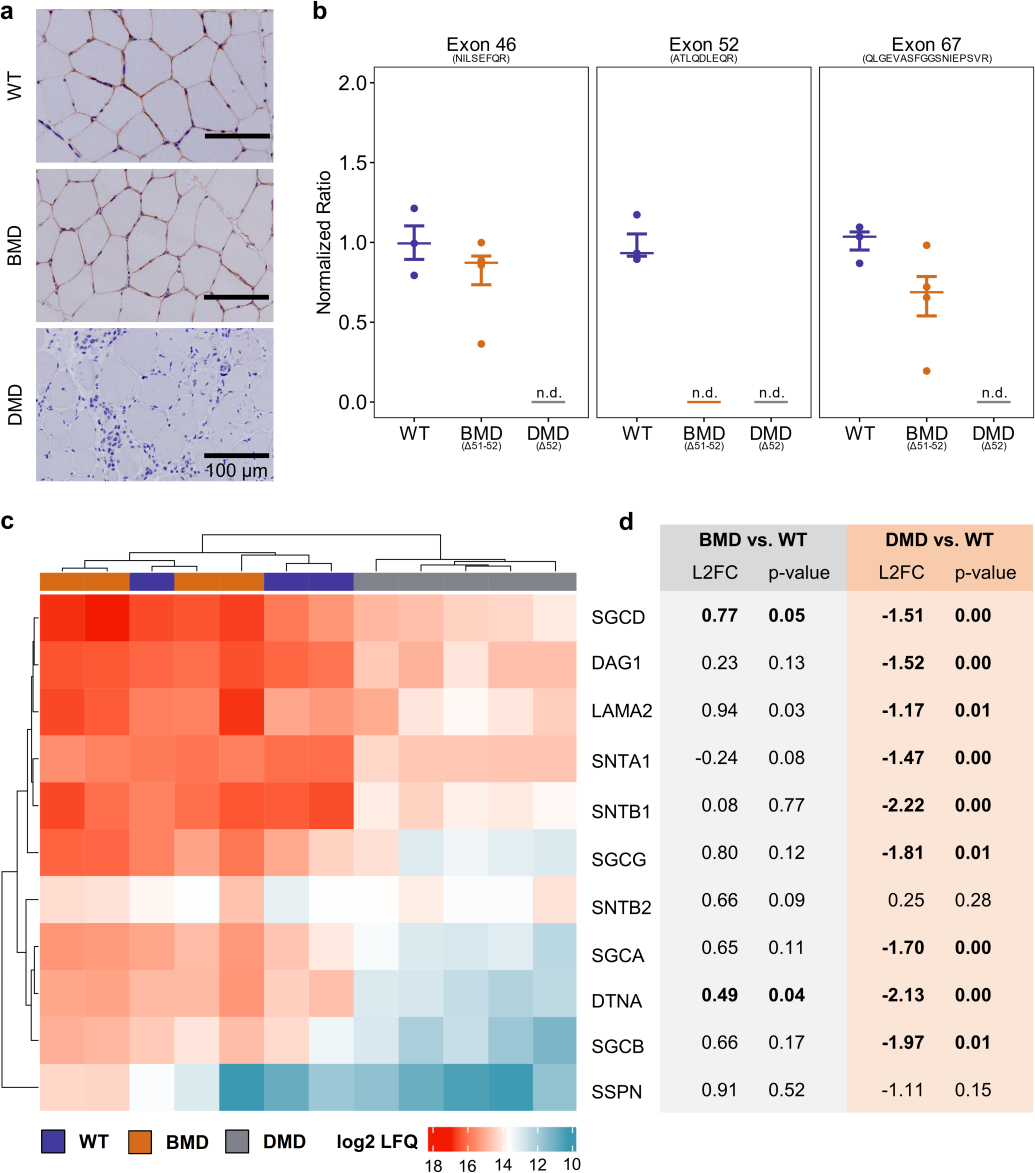
Abundances of dystrophin and proteins of the dystrophin‐glycoprotein complex (DGC). (a) Representative sections of *M. fibularis tertius* samples from WT, BMD, and DMD pigs after immunohistochemical staining of dystrophin using the DYS1‐antibody. (b) Quantification of dystrophin‐specific peptides in muscle samples from WT, BMD and DMD pigs using mass spectrometry. Dystrophin peptide levels are normalized using heavy labelled peptides. The ratios were calculated in reference to the mean of WT levels. Peptide levels in BMD vs. WT samples were compared using T‐tests. (c) Heat map of log2 label‐free quantification intensity values of proteins of the DGC. Dendrograms of rows and columns were calculated with unsupervised clustering. (d) Corresponding log_2_ fold changes (L2FC) and results of T‐tests comparing the levels of DGC proteins in BMD and DMD samples with WT samples. Abbreviations: DAG1 = dystroglycan; DTNA = dystrobrevin alpha; LAMA2 = laminin subunit alpha 2; SGCA = sarcoglycan alpha; SGCB = sarcoglycan beta; SGCD = sarcoglycan delta; SGCG = sarcoglycan gamma; SNTA1 = syntrophin alpha 1; SNTB1 = syntrophin beta 1; SNTB2 = syntrophin beta 2; SSPN = sarcospan.

To demonstrate the presence of dystrophin protein in the skeletal muscle of pigs with an intact *DMD* reading frame, a cross‐reactive antibody against human dystrophin targeting the rod domain was used (NCL‐DYS1, Leica Biosystems). Dystrophin was only localized at the sarcolemma of WT and BMD muscles (Figure 7a). Targeted quantification of dystrophin‐specific peptides coded by *DMD* exons 46, 52 and 67 confirmed the complete absence of dystrophin in DMD muscle and the lack of the exon 52–encoded peptide sequence in BMD muscle. The exon 46– and exon 67–encoded dystrophin peptides were, as a tendency, but not significantly, reduced in BMD vs. WT samples (Figure 7b). The abundances of proteins from the dystrophin‐glycoprotein complex (DGC) were extracted from the holistic proteome analysis and are displayed in Figure 7c. In DMD muscle, multiple DGC proteins were significantly reduced in abundance, whereas the DGC protein profile of BMD muscle was similar to the WT samples. Consistent with previous analysis of triceps muscle, the proteome profiles of fibularis tertius muscle (Figure S5) differed markedly between DMD pigs and WT controls, whereas the proteome profile of BMD samples was very similar to the WT proteome profiles.

## 4 Discussion

The results of our present study complement earlier research on our BMD [20] and DMD pig models [15] and confirm that our breeding cohort effectively produces offspring with stable, phenotypically reliable traits that rapidly and consistently mimic human disease phenotypes. In addition to the established methods for characterizing dystrophic muscle in pig models, the current study performed the first in vivo quantification of skeletal muscle force and function in different dystrophic (DMD and BMD) and nondystrophic (WT) pig littermates. By implementing this method systematically, we detected relevant differences between our experimental groups and observed a high degree of consistency among individual animals of a group.

The functional muscle analysis can be broadly categorized into temporal and force parameters. Two main contraction types—the single‐peak twitch contraction and the stronger, longer‐lasting tetanic contraction—have been analysed. In DMD animals, the twitch contraction cycle was prolonged in both contraction and relaxation time. Although the tetanic cycle is less optimal for evaluating time parameters because of its prolonged plateau phase resulting from multiple stimulations, a prolonged relaxation time in the DMD model was also observed in this contraction type. The prolonged relaxation time in DMD pigs mirrors increased passive muscle stiffness because of the dystrophic alterations consistently observed by histopathological and proteomic investigations and supported by the serum marker analysis. Importantly, both the BMD and the WT control groups exhibited similar results in contractile function.

In terms of absolute and relative force, DMD and—to a lesser extent—BMD animals displayed substantial muscle weakness in both twitch and tetanic contractions. The reduced muscle strength in dystrophic pigs was also evident in the examination of maximum and average contraction and relaxation rates. Interestingly, BMD animals showed intermediate values between the DMD and WT genotypes, indicating that the expression of a shortened dystrophin has a beneficial effect, but does not completely restore normal muscle function.

Of note, the functional muscle assessment was for the first time able to discriminate BMD pigs from WT pigs at the age of 3.5 months, as so far, no significant differences were detected in other tests such as blood chemistry, histopathology or in vivo functional measurements, including echocardiography and behavioural testing [20]. Despite the successful improvement of the clinical phenotype by *DMD* reading frame correction, there are still notable limitations in muscle physiology, which can be detected by sensitive techniques, such as the used muscle force analysis. Previous studies in BMD mouse models [31, 32] and *mdx*52 mouse models applying exon‐skipping strategies [33, 34, 35] have reported heterogeneous outcomes regarding dystrophin restoration and functional improvement. Although several findings align with our observations, others diverge. These discrepancies may stem from differences in the targeted exons, treatment protocols, delivery efficiencies and evaluation methodologies. Additionally, species‐specific factors—such as muscle size, regenerative capacity and immune response—may influence outcomes. Notably, the substantially greater body weight and corresponding mechanical load on muscle fibres in pigs create more physiologically demanding conditions. These factors increase the stringency and enhance the translational relevance of the porcine model used in this study.

A possible explanation for the reduced muscle strength of BMD pigs is the expression of an internally shortened dystrophin. The loss of exons 51 and 52 eliminates the coding sequence for 117 amino acids in the rod domain of dystrophin and also affects the third interspersed hinge [20]. Interestingly, different dystrophin products produced by exon 51 skipping repair displayed differences in stability, structure and lipid binding properties [36], which may also affect their function in vivo. Given that neither immunofluorescence‐based fibre typing nor proteomic profiling indicated significant differences between BMD and WT muscles, myofiber composition is unlikely to be a major determinant of the observed functional deficits.

The functional findings were supported by histological evaluation and also the proteomics analyses of the stimulated muscle groups, demonstrating highly dystrophic muscle tissue in DMD pigs, whereas the skeletal muscle architecture and the abundances of dystrophin and dystrophin‐associated proteins in BMD samples were similar to WT tissue. These results are in line with previously described findings in the *Mm. triceps brachii* and *biceps femoris* and substantiate the hypothesis that DMD muscle degeneration manifests uniformly across multiple muscle groups [20].

Our results emphasize the importance and reliability of quantitative in vivo skeletal muscle function analysis for future studies. The method offers high sensitivity across the spectrum of functional muscle physiology and provides a wide range of parameters, including temporal dynamics, force kinetics and fatigue. Integrating functional muscle assessments into therapeutic studies, comparing treated and nontreated groups, not only in DMD but also in other musculoskeletal diseases, will enhance the sensitivity for detecting treatment effects. Thus, it will be interesting to see how larger gene editing approaches (e.g., deletion of exons 45–55 [37]), expression of mini‐ or microdystrophins [38] or treatment with steroids [39] affect the muscle function of DMD pigs. Since these measurements can be performed in vivo, disease progression can be monitored over time. This allows for longitudinal assessments, which provide valuable insights into disease dynamics, improve the detection of treatment effects and reduce variability by using the same subjects for repeated measurements. Consequently, this approach also enhances animal welfare by reducing the number of animals needed for experiments.

### 4.1 Limitations of the Study

In order to overcome limitations associated with small group sizes in large animal experiments, standardization is essential to enhance reliability [40]. In the present study, full‐ or half‐siblings raised together were used to standardize both genetic background and environment. Using anaesthetized pigs allowed objective measurements of skeletal muscle function independent of individual motivation and endurance. Furthermore, all tests were performed by the same study team to ensure consistency and further minimize experimental variation. Consequently, despite the relatively small group sizes in our present study, we were able to detect several statistically significant and biologically relevant differences among genotypes. Since—because of initial technical difficulties—the fatigue data could not be used from all animals, a statistical evaluation was not possible. Nevertheless, the fatigue data revealed an obvious difference between DMD vs. BMD and WT pigs.

## Conflicts of Interest

Michaela Blasi, Hristiyan Hristov, Martin Kraetzl, Sonja Fiedler, Elisabeth Kemter, Mayuko Kurome, Barbara Kessler, Valeri Zakhartchenko, Christian Kupatt, Nikolai Klymiuk, Andreas Blutke, Michael Stirm, Florian Jaudas and Eckhard Wolf declare no conflicts of interest. Maggie C. Walter has served on advisory boards for Avexis, Biogen, Novartis, Pfizer, Roche, Santhera, Sarepta, Pharnext, PTC Therapeutics, Ultragenyx and Wave Sciences; received funding for travel or speaker honoraria from Avexis, Biogen, PTC Therapeutics, Ultragenyx, Santhera and Sarepta; and worked as an ad hoc consultant for AskBio, Audentes Therapeutics, Avexis, Biogen Pharma GmbH, Fulcrum Therapeutics, GLG Consult, Guidepoint Global, Gruenenthal Pharma, Novartis, Pharnext, PTC Therapeutics and Roche.

## Supporting information


**Figure S1:** Weight differences between genotypes. Body mass of pigs on the day of investigation. Means ± SEM and values of individual animals are shown. Statistical significance was assessed using one‐way ANOVA with Turkey's multiple comparisons test (***p < 0.001).
**Figure S2:** Relative force over a range of stimulation frequencies. Electrical stimulation impulses were gradually increased, and the resulting force generated by the muscle was measured to assess its contractile properties. Groups are labelled by different colours as in Figure S1.
**Figure S3:** Original trajectories. Original graphs of the course of a tetanic contraction in WT, BMD and DMD as displayed by the Aurora device. Note the different scales of the *y*‐axis.
**Figure S4:** Endurance of DMD, BMD and WT muscles. (a) Decline in force (% of initial peak) over 100 consecutive stimulations. The force generated during the first contraction cycle (strongest) is set to 100%. The slope of the curve represents the rate of force reduction, indicating fatigue. (b) Number of stimulations required to reduce force output to 75% of the initial contraction force. Means ± SEM and values of individual animals are presented.
**Figure S5:** Holistic proteome analysis. (a–d) Proteomic analysis of skeletal muscle samples from tibialis cranialis muscle samples of WT, BMD and DMD pigs. Unsupervised hierarchical clustering of differentially abundant proteins (a) and principal component analysis (b). Visualization of proteome changes in DMD vs. WT samples (c) and BMD vs. WT samples (d) using volcano plots, with the permutation‐based false discovery rate (FDR) significance cutoff represented by the black curves.
**Table S1:** Measurement protocol. Overview table of the exact measurement protocol used to obtain the muscle strength data for the individual animals.

## Data Availability

Data and materials are available upon request from the corresponding author.
