# Supplementary material for: Reduced Muscle Force in Dystrophic DMDΔ52 Pigs Is Incompletely Restored by Systemic Transcript Reframing (DMDΔ51–52)
Source: J Cachexia Sarcopenia Muscle. 2025 Oct 1;16(5):e70084. doi: 10.1002/jcsm.70084 (PMC12485293; doi:10.1002/jcsm.70084)
Supplement: Supplementary file 1 — Figure S1: Weight differences between genotypes. Body mass of pigs on the day of investigation. Means ± SEM and values of individual animals are shown. Statistical significance was assessed using one‐way ANOVA with Turkey's multiple comparisons test (***p < 0.001). Figure S2: Relative force over a range of stimulation frequencies. Electrical stimulation impulses were gradually increased, and the resulting force generated by the muscle was measured to assess its contractile properties. Groups are labelled by different colours as in Figure S1. Figure S3: Original trajectories. Original graphs of the course of a tetanic contraction in WT, BMD and DMD as displayed by the Aurora device. Note the different scales of the y‐axis. Figure S4: Endurance of DMD, BMD and WT muscles. (a) Decline in force (% of initial peak) over 100 consecutive stimulations. The force generated during the first contraction cycle (strongest) is set to 100%. The slope of the curve represents the rate of force reduction, indicating fatigue. (b) Number of stimulations required to reduce force output to 75% of the initial contraction force. Means ± SEM and values of individual animals are presented. Figure S5: Holistic proteome analysis. (a–d) Proteomic analysis of skeletal muscle samples from tibialis cranialis muscle samples of WT, BMD and DMD pigs. Unsupervised hierarchical clustering of differentially abundant proteins (a) and principal component analysis (b). Visualization of proteome changes in DMD vs. WT samples (c) and BMD vs. WT samples (d) using volcano plots, with the permutation‐based false discovery rate (FDR) significance cutoff represented by the black curves. Table S1: Measurement protocol. Overview table of the exact measurement protocol used to obtain the muscle strength data for the individual animals. [file JCSM-16-e70084-s001.pdf]

Supplementary Information for

Reduced Muscle Force in Dystrophic *DMD*Δ52 Pigs is Incompletely Restored by Systemic Transcript Reframing (*DMD*Δ51-52)

Michaela Blasi, Hristiyan Hristov, Jan B. Stöckl, Martin Kraetzl, Sonja Fiedler, Elisabeth Kemter, Mayuko Kurome, Barbara Kessler, Josep M. Cambra, Valeri Zakhartchenko, Maggie C. Walter, Christian Kupatt, Nikolai Klymiuk, Thomas Fröhlich, Andreas Blutke, Michael Stirm, Florian Jaudas, Eckhard Wolf*

*correspondence: ewolf@genzentrum.lmu.de

This PDF file includes:

Figures S1 to S5

Table S1

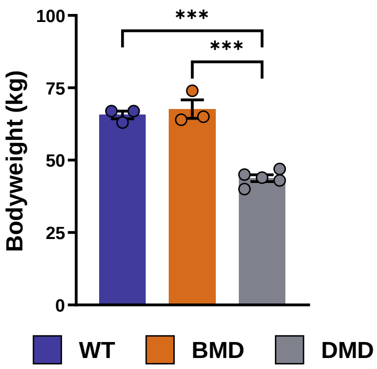

Fig. S1 Weight differences between genotypes Body mass of pigs on the day of investigation. Means ± SEM and values of individual animals are shown. Statistical significance was assessed using one-way ANOVA with Turkey's multiple comparisons test (***p < 0.001).

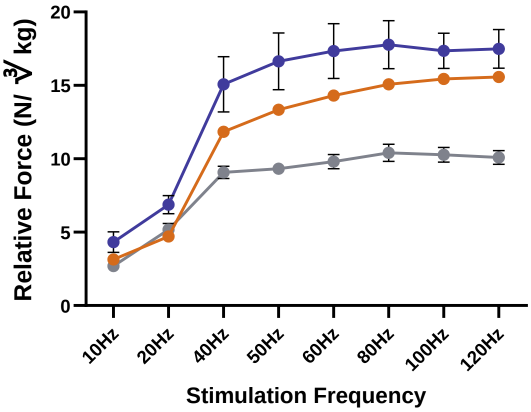

Fig. S2 Relative force over a range of stimulation frequencies Electrical stimulation impulses were gradually increased, and the resulting force generated by the muscle was measured to assess its contractile properties. Groups are labeled by different colors as in Fig. S1.

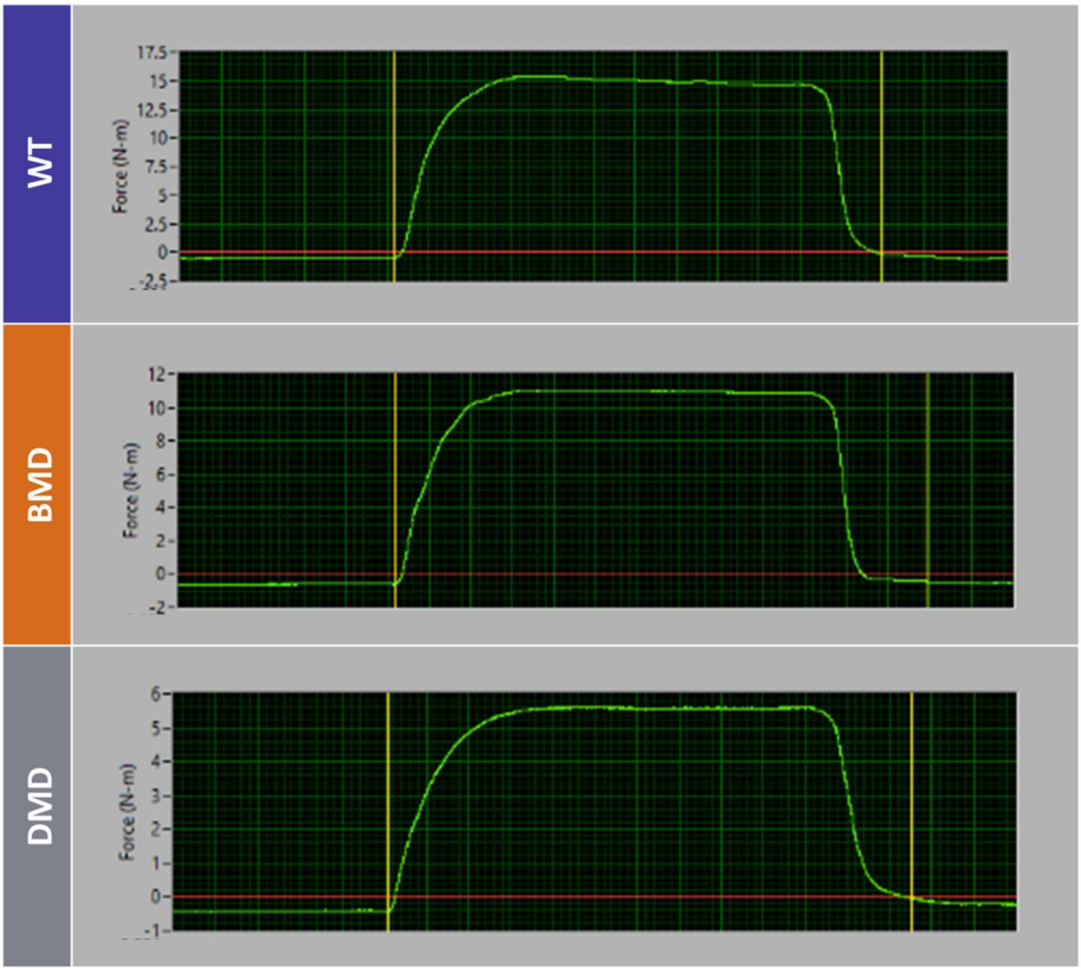

Fig. S3 Original trajectories Original graphs of the course of a tetanic contraction in WT, BMD and DMD as displayed by the Aurora device. Note the different scales of the y-axis.

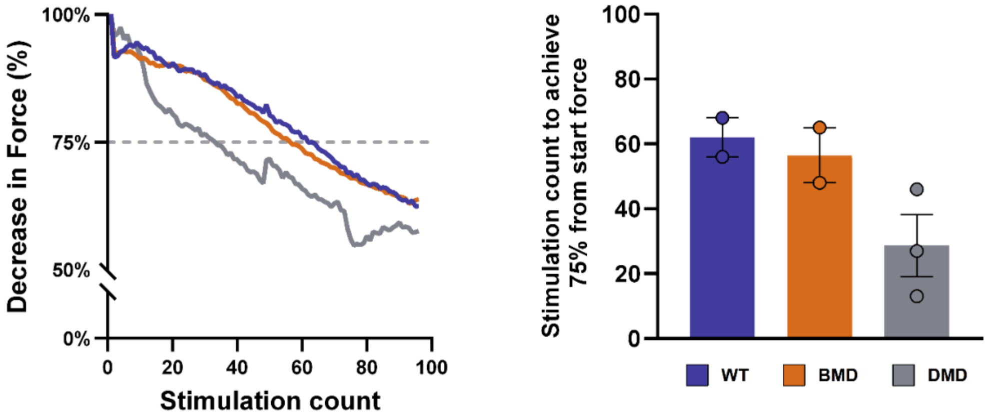

Fig. S4 Endurance of DMD, BMD and WT muscles (**a**) Decline in force (% of initial peak) over 100 consecutive stimulations. The force generated during the first contraction cycle (strongest) is set to 100%. The slope of the curve represents the rate of force reduction, indicating fatigue. (**b**) Number of stimulations required to reduce force output to 75% of the initial contraction force. Means ± SEM and values of individual animals are presented.

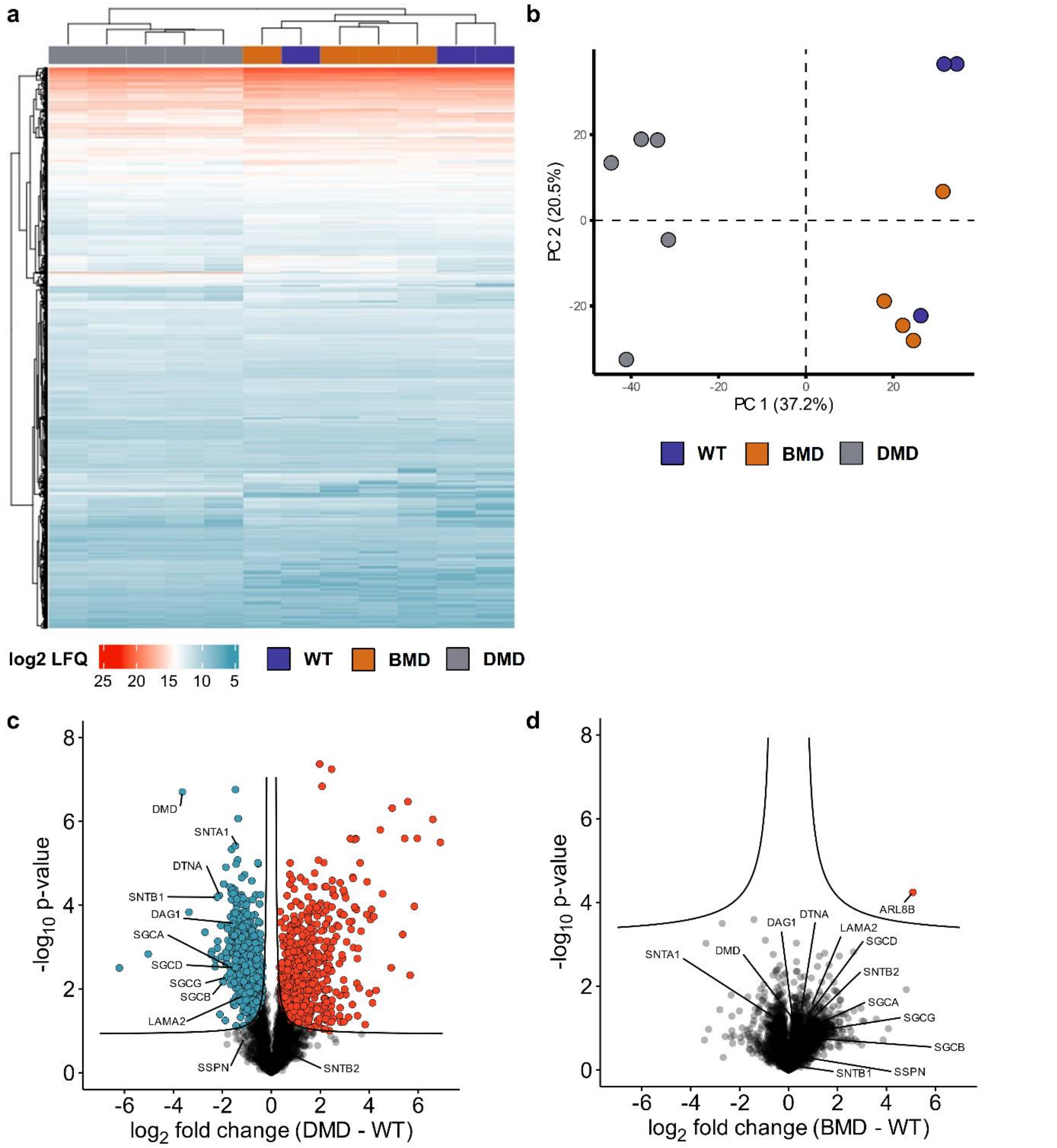

Fig. S5 Holistic proteome analysis (**a-d**) Proteomic analysis of skeletal muscle samples from tibialis cranialis muscle samples of WT, BMD, and DMD pigs. Unsupervised hierarchical clustering of differentially abundant proteins (**a**) and principal component analysis (**b**). Visualization of proteome changes in DMD vs. WT samples (**c**) and BMD vs. WT samples (**d**) using volcano plots, with the permutation-based false discovery rate (FDR) significance cutoff represented by the black curves.

Measurement	Amount	Frequency [Hz]	Stimulus time / Puls width [ms]	Interstimulus interval [s]	Purpose
**Twitch**<br>*Resting (1 min)*	5x	-	0.2	15	electrode adjustment
**Tetanus**<br>*Resting (3 min)*	2x	100	500	15	
**Twitch**<br>*Resting (1min)*	5x	-	0.2	15	data collection
**Tetanus**<br>*Resting (3 min)*	5x	100	500	15	
**Force Frequency**<br>*Resting (5 min)*	8x	10 -120	500	60	
**Fatigue**	100x	60	500	5	

Table S1 Measurement protocol Overview table of the exact measurement protocol used to obtain the muscle strength data for the individual animals.
